# Structural Transformations of Hydrolysates Obtained from Ti-, Zr-, and Ti, Zr-Solutions Used for Clay Pillaring: Towards Understanding of the Mixed Pillars Nature

**DOI:** 10.3390/ma12010044

**Published:** 2018-12-24

**Authors:** Krzysztof Bahranowski, Agnieszka Klimek, Adam Gaweł, Katarzyna Górniak, Alicja Michalik, Ewa Serwicka-Bahranowska

**Affiliations:** 1 Faculty of Geology, Geophysics and Environmental Protection, AGH University of Science and Technology, al. Mickiewicza 30, 30-059 Krakow, Poland; agaklimek@o2.pl (A.K.); agawel@agh.edu.pl (A.G.); gorniak@agh.edu.pl (K.G.); 2 Jerzy Haber Institute of Catalysis and Surface Chemistry, Niezapominajek 8, 30-239 Krakow, Poland; ncmichal@cyf-kr.edu.pl (A.M.); ncserwic@cyf-kr.edu.pl (E.S.-B.)

**Keywords:** hydrolysis, titania, zirconia, zirconium titanate, pillared clays

## Abstract

Structural characteristics of hydrolysates formed from the aqueous Ti-, Zr-, and Ti, Zr-pillaring solutions prepared from inorganic precursors (TiCl_4_ and ZrOCl_2_), was investigated and compared with that of precipitates obtained from the same solutions after a slight alkalization of pH to the values reported for the conditions of clay pillaring. The materials were recovered by lyophilization and subsequently subjected to calcination at 500, 800 and 1000 °C. Of special interest was the effect of pH on the possible formation of mixed Ti, Zr-oxide species. Powder X-ray diffraction (XRD), Raman spectroscopy and scanning electron microscope/energy dispersive spectrometer (SEM/EDS) analysis showed that even a relatively moderate alteration of pH in Ti-, Zr-, or Ti, Zr-precursor solutions caused substantial changes in the outcome of hydrolytic transformations, manifested by different phase and/or chemical composition of the resulting hydrolysates. Analysis of thermal evolution of hydrolysates showed that alkalization facilitated the transformation of anatase into rutile in materials obtained from Ti-pillaring solution, but retarded tetragonal to monoclinic zirconia conversion in samples derived from Zr-pillaring agent. The most striking effect was observed for the mixed Ti, Zr-pillaring solution, where an increase of pH enabled the formation of zirconium titanate as the only crystalline phase, rather than a multiphase mixture of anatase, monoclinic zirconia and zirconium titanate obtained from the more acidic precursor. The finding supports the model of mixed Ti-O-Zr network in props generated in Ti, Zr-pillared montmorillonites.

## 1. Introduction

Pillaring of clays is a process in which common interlayer hydrated cations compensating the layer charge are exchanged with large hydrated inorganic polymeric oxy-hydroxy cationic species which prop open the silicate layers [1]. Upon high temperature treatment, the oligomers are transformed into oxide nanoparticles and link permanently the neighboring layers. The resulting solids, referred to as pillared interlayered clays (PILCs), are characterized by large surface area, high pore volume and pore size tunable from micropore to low mesopore range. In addition, appropriate modification of the chemical nature of pillars enables control of the materials acid–base and redox properties. In recent work, we have shown that pillaring of montmorillonite clay with mixed [Ti, Zr]-pillaring solution yielded material of unique structural, textural and surface acid–base properties, differing from those of reference Ti-PILC and Zr-PILC structures [2]. As a result, the [Ti, Zr]-PILC displayed superior properties when used as support for Pd and/or Cr catalysts for the destruction of chlorinated volatile organic compounds [3]. The study suggested that pillars in [Ti, Zr]-PILC consisted of nano-clusters of a quasi-amorphous Ti-Zr mixed oxide, rather than separate titania and zirconia pillars, of short-range order similar to that of zirconium titanate phase. However, it should be noted that there is a lack of a general agreement as to the nature of props formed from pillaring agents containing both the Ti and the Zr precursor. Thus, although Das, et al. [4] reached a similar conclusion as to the binary nature of Ti-Zr pillars, some other reports discussed the results of smectite pillaring with mixed Ti-Zr pillaring solutions in terms of separate TiO_2_ and ZrO_2_ pillars [5,6]. 

The nature of the final form of intercalated species depends on the hydrolytic transformations occurring in pillaring solutions, which, in turn, are pH dependent, since an increase of pH facilitates the hydrolysis [7]. We have reported that during preparation of mixed component [Ti, Zr]-PILC and reference one-component Ti-PILC, Zr-PILC, after mixing of pillaring solutions with the aqueous suspension of clay, the final pH of the suspensions stabilized at a value higher than that of the corresponding pillaring agent, i.e., it increased from from 0.1 to 1.2 for Ti-based system, from 1.0 to 1.5 for Zr-based system, and from 0.6 to 1.2 for Ti, Zr-based system, [2]. Hydrolytic transformations occurring in the pillaring solutions upon pH modification are bound to have an impact on the final effect of pillaring therefore, we decided to get insight into the relation between the pH of pillaring agents and the nature of hydrolysates obtained from such precursors. To achieve this aim, in the present work, we investigated the character of precipitates formed from the as received Ti-, Zr-, and Ti, Zr-pillaring agents (lower pH values), and compared their characteristics with those of precipitates obtained from the same solutions after adjustment of pH to the values observed during pillaring. Of special interest was the effect of pH on the possible formation of mixed [Ti, Zr] oxide species, because an understanding of the nature of pillars in Ti, Zr-pillared clays is of key importance for the interpretation of their catalytic, photocatalytic and sorption properties. However, the significance of this study is not limited to the chemistry of pillared clays, since zirconium titanate-based systems are not only fascinating catalytic materials in their own right, but find also use in advanced microwave technology, optical devices, and as ceramic insulators [8,9,10,11,12]. 

## 2. Materials and Methods

### 2.1. Materials

In the present work TiCl_4_ (Aldrich, pure, ≥98%, St. Louis, MO, USA) and ZrOCl_2_·8H_2_O (Fluka, analytical grade, Munich, Germany) were used for the preparation of Ti, Zr or [Ti, Zr]-containing solutions, following the preparative procedure of pillaring agents described in [2]. Briefly, Ti-pillaring solution was prepared by theslow addition of 4.5 mL TiCl_4_ to 1.25 mL 6 M HCl under vigorous stirring, followed by dilution with 50 mL distilled water. The stirring continued at room temperature for 3 h. The pH value of the resulting clear solution was 0.1. Zr-pillaring solution was obtained by dissolving 13.5 g ZrOCl_2_·8H_2_O in 333 mL distilled water and stirring for 1 h at room temperature. The pH value of the clear solution was 1.0. Mixed [Ti, Zr] solution was prepared by the dropwise addition of Ti-pillaring agent to the Zr-containing solution, to obtain 1:1 molar ratio of Ti and Zr. After 1 h stirring at room temperature the pH of the clear solution was 0.6.

Two series of experiments were carried out with the prepared pillaring solutions. One involved obtaining the precipitates from the freshly prepared Ti-, Zr-, and Ti, Zr-pillaring agents by lyophilization. These samples are denoted Ti-0.1, Zr-1.0, and Ti, Zr-0.6 (numerical indices correspond to the natural pH of the solutions), and after 3 h calcination as Ti-0.1-temp, Zr-1.0-temp, and Ti, Zr-0.6-temp, where temp is the calcination temperature (500, 800 or 1000 °C). The other series was prepared from the same pillaring agents, but prior to lyophilization their pH was adjusted with 0.1 M NH_3_aq to the values registered after mixing of the pillaring solutions with the suspension of montmorillonite. Thus, obtained hydrolysates are referred to as Ti-1.2, Zr-1.5, and Ti, Zr-1.2 (numerical indices correspond to the ammonia adjusted pH of the solutions), and after 3 h calcination as Ti-1.2-temp, Zr-1.5-temp, and Ti, Zr-1.2-temp, where temp is the calcination temperature (500, 800 or 1000 °C).

### 2.2. Methods

Powder X-ray diffraction (XRD) patterns were recorded with Rigaku SmartLab diffractometer (Neu-Isenburg, Tokyo, Japan) under the following conditions: Graphite-monochromatized CuKα radiation, operating voltage 45 kV, current 200 mA, step size 0.05° and counting time 1s/step. 

Raman spectra were collected with a DXR Raman microscope (Thermo Scientific, Waltham, MA, USA) using 532 nm excitation laser wavelength, power level 6 mW and spectral resolution 2 cm^−1^.

Field emission scanning electron microscopy (FESEM) studies were carried out for the uncoated samples with the use of an FEI Quanta 200 FEG SEM (Hillsboro, OR, USA) equipped with a secondary electron (SE) and back-scattered electron (BSE) detectors. Energy dispersive spectrometer (EDS, FEI Quanta, Hillsboro, OR, USA) was employed to monitor variations of chemical composition in mixed Ti, Zr phases, using polished specimens of samples embedded in epoxy resin.

## 3. Results and Discussion

### 3.1. Hydrolysates Formed fromTi-Pillaring Solution

**Figure 1:**
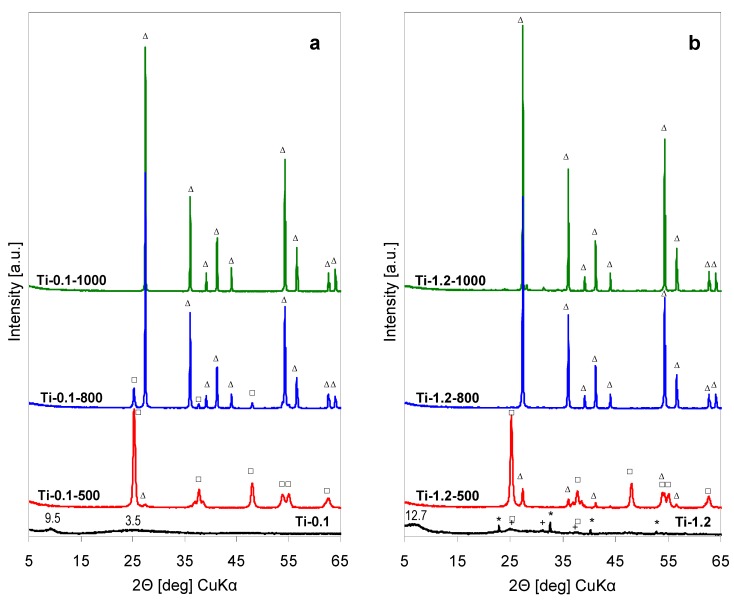
Powder X-ray diffraction (XRD) patterns illustrating the effect of thermal treatment on hydrolysates obtained by lyophilization of Ti-pillaring solution: (**a**) As received at pH = 0.1, (**b**) after pH adjustment to 1.2 (□ anatase, ∆ rutile, + brookite, * NH_4_Cl).

The XRD patterns of precipitates obtained by lyophilization of the as received Ti-pillaring solution (pH = 0.1) are presented in Figure 1a, while Figure 1b shows XRD diagrams of lyophilized precipitates obtained from solutions whose pH has been adjusted to 1.2, the value observed upon preparation of Ti-pillared montmorillonite. The XRD pattern of the as received lyophilized Ti-0.1 sample shows only a broad reflection around 2Θ≈ 9.3°, corresponding to ca. 9.5 Å interplanar distance, and an even broader, low intensity bump centered around 2Θ ≈ 25° (d ≈ 3.5 Å), which points to the chiefly amorphous character of the solid components. The low angle reflection may be taken as an indication that part of the amorphous solid tends to form a layered structure, e.g., related to the lepidocrocite-type protonated titanate [13]. The feature with d ≈ 3.5 Å may be attributed to an amorphous phase from which a titania polymorph is going to nucleate. After calcination of the precipitate at 500 °C (sample Ti-0.1-500) the XRD pattern shows a set of intense reflections characteristic of crystalline anatase (A), accompanied by trace amounts of rutile (R). Material calcined at 800 °C contains a higher relative share of rutile, and after heat treatment at 1000 °C, rutile is the only detected crystalline TiO_2_ phase. 

The XRD pattern of Ti-1.2 precipitate obtained from pillaring solution whose pH was raised with NH_3_aq to the value observed during Ti-PILC formation, shows that some ammonium chloride has been formed in the process, as indicated by the set of sharp reflections characteristic of this salt. The broad reflection at 2Θ ≈ 7° (d ≈ 12.5 Å) indicates that also here an amorphized layered structure is formed, probably of similar nature as in Ti-0.1, but with higher content of water in the interlayer [13]. The titania-related phase, although still poorly crystalline, is better defined than in the case of Ti-0.1 sample. The most pronounced reflection around 2Θ ≈ 25° (d ≈ 3.5 Å) and the reflection at 2Θ ≈ 37.5° (d ≈ 2.39 Å) suggests nucleation of anatase and/or brookite. A contribution of the latter may be inferred from the faint reflection around 2Θ ≈ 31° (d ≈ 2.88 Å) diagnostic of brookite polymorph. XRD pattern of Ti-1.2-500 sample shows that after calcination at 500 °C the material contains predominantly anatase, but transformation to rutile is more advanced than in the Ti-0.1-500 counterpart. Rutile is the only crystalline phase found in samples calcined at 800 and 1000 °C. Thus, a comparison of the thermal evolution of both precipitates shows that transformation of anatase into rutile is slightly facilitated in the material obtained from alkalized pillaring solution.

**Figure 2:**
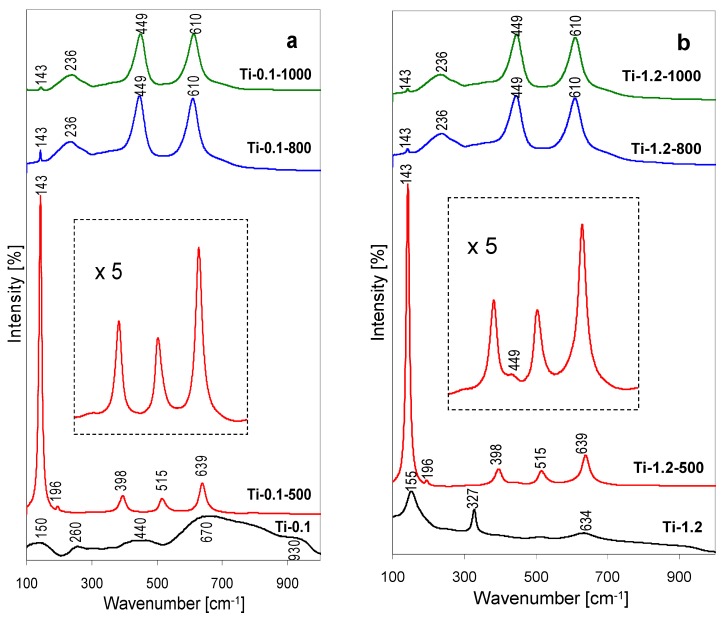
Raman spectra illustrating the effect of thermal treatment on hydrolysates obtained by lyophilization of Ti-pillaring solution: (**a**) As received at pH = 0.1, (**b**) after pH adjustment to 1.2.

Structure of the precipitates was also studied with Raman spectroscopy, more powerful than XRD for detecting short range order in amorphous nanodomains [14]. Therefore, Raman analysis was expected to shed more light on the structure of the thermally untreated Ti-0.1 and Ti-1.2 precipitates, essentially amorphous from the XRD point of view. Various titania polymorphs and layered titanates are known to exhibit characteristic Raman spectra, and the present interpretation of the data follows previous assignments [15,16,17,18]. The Raman spectra monitoring thermal evolution of Ti-0.1 precipitate, are shown in Figure 2a, those obtained for the Ti-1.2 series, in Figure 2b.

The Raman spectrum of the lyophylized Ti-0.1 sample, shown in Figure 2a, displays several ill-defined broad bands, centered around 150, 260, 440, 670, 930 cm^−1^. All, except the first one, may be considered as envelopes of broadened bands stemming from amorphized lepidocrocite-type titanate phase [18], whose presence has been suggested on the base of the XRD pattern (Figure 1a). The feature around 150 cm^−1^ appears in the range where the most intense bands of anatase or brookite appear, and is attributed to the amorphous titania structures responsible for the broad XRD bump centered at d = 3.5 Å in Figure 1a. 

The spectrum of Ti-1.2 precipitate, obtained from the pillaring solution after pH adjustment, shows better resolved features. The set of bands at 155, 327 and 634 cm^−1^ points to the formation of poorly crystalline brookite [16,17], thus substantiating the tentative assignment derived from the XRD data. Indeed, Pottier, et al. [19] reported that Ti(OH)_2_(Cl)_2_(OH_2_)_2_ species formed during hydrolysis of TiCl_4_, in strongly acidic medium enriched with chloride ions, initially tend to form the brookite phase. In this spectral range the dominant band of NH_4_Cl appears around 180 cm^−1^, but the amount of impurity is too small to make an impact on the recorded Raman spectrum. The spectra obtained for both series of thermally treated samples essentially agree with the conclusions drawn from XRD examination. Thus, the spectra of samples after calcination at 500 °C are dominated by the bands at 143, 196, 398, 515 and 639 cm^−1^ characteristics of anatase [15]. The presence of rutile admixture can be detected in Ti-1.2-500 as a shoulder at 449 cm^−1^, clearly visible in the magnified part of the spectrum in Figure 2b. The trace of rutile indicated by XRD in Ti-0.1-500 is apparently too small to reveal its presence in the Raman spectrum (magnified fragment in Figure 2a). The spectra of samples calcined at higher temperatures are dominated by the bands typical of rutile, i.e., a weak one at 143 cm^−1^ and three strong features at 236, 449 and 610 cm^−1^, which agrees with the XRD analysis pointing to the occurrence of anatase to rutile phase transformation [15]. The presence of some anatase remnants, revealed by XRD in sample Ti-0.1-800, may be inferred from the slightly higher, than in other samples, intensity of the 143 cm^−1^ band, which indicates a contribution from the coincident most intense band of anatase (Figure 2a).

**Figure 3:**
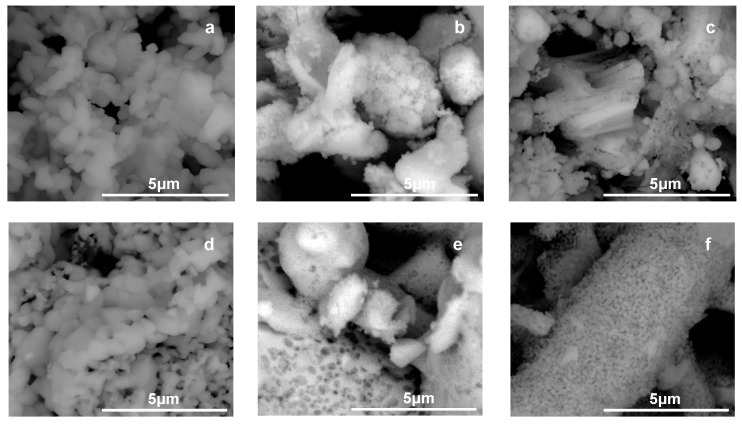
Field Emission Scanning Electron Microscope (FESEM) images of: (**a**) Ti-0.1-1000, (**b**) Zr-1.0-1000, (**c**) Ti, Zr-0.6-1000, (**d**) Ti-1.2-1000, (**e**) Zr-1.5-1000, (**f**) Ti, Zr-1.2-1000.

SEM images of samples Ti-0.1-1000 and Ti-1.2-1000 presented in Figure 3a,d, respectively, show that after calcination at 1000 °C the morphology of both materials is quite similar, with particles size being, in general, ≤0.5 μm.

### 3.2. Hydrolysates Formed from Zr-Pillaring Solution

**Figure 4:**
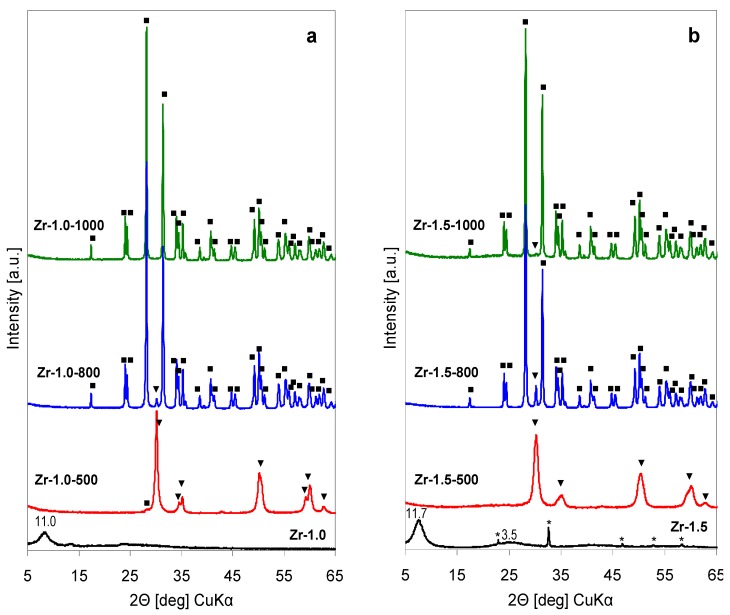
XRD patterns illustrating the effect of thermal treatment on hydrolysates obtained by lyophilization of Zr- pillaring solution: (**a**) As received at pH = 1.0, (**b**) after pH adjustment to 1.5 (▼ *t*-ZrO_2_, ■ *m*-ZrO_2_, * NH_4_Cl).

Figure 4a shows the XRD patterns of precipitates obtained from the as received Zr-pillaring solution (pH = 1.0), Figure 4b gathers XRD data obtained for the solids prepared from the Zr-pillaring agent whose pH has been raised to 1.5, the value observed during Zr-PILC synthesis. The XRD pattern of the as received lyophilized Zr-1.0 sample appears essentially amorphous. It is dominated by a broad reflection around 2Θ ≈ 8°, corresponding to ca. 11 Å interplanar distance. The most intense reflections of various ZrOCl_2_ hydrates appear in the 7° < 2Θ < 8.6° range (e.g., ZrOCl_2_·8H_2_O, JCPDS 00-032-1498, ZrOCl_2_·6H_2_O, JCPDS 00-047-0815, ZrOCl_2_·4H_2_O, JCPDS 00-018-1497). Matsui and Ohgai [20], in their extensive study of ZrOCl_2_ hydrolysis, referred to such a product as chlorine-containing hydrous zirconia. Calcination of this precipitate at 500 °C leads to crystallization of tetragonal ZrO_2_ polymorph (*t*-ZrO_2_). In addition, in the XRD pattern of Zr-0.1-500 a trace of peak at 2Θ = 28.2° (d = 3.162 Å), corresponding to the most intense reflection of the monoclinic zirconia (*m*-ZrO_2_), is observed, which shows that a small degree of transformation of the metastable *t*-ZrO_2_ to the stable *m*-ZrO_2_ has already occurred. After calcination at 800 °C the transformation is almost complete and the material is composed predominantly of *m*-ZrO_2_, with only a trace of unreacted tetragonal phase, manifested by the remainder of the most intense reflection of *t*-ZrO_2_ at 2Θ = 30.2° (d = 2.957 Å). Sample calcined at 1000 °C contains only *m*-ZrO_2_. 

The XRD pattern of Zr-1.5 precipitate obtained from pillaring solution whose pH was adjusted to 1.5 with ammonia, shows that, similarly as in the case of Ti-1.2, some ammonium chloride has been formed. Otherwise, the diffractogram displays broad reflection at 2Θ ≈ 7.5° (d ≈ 11.7 Å), together with a broad bump centered around 2Θ ≈ 25° (d ≈ 3.5 Å), i.e. at a lower angle than that expected for amorphous ZrO_2_ (broad envelope around 2Θ = 30 °C [21]). This shows that, even after an increase of pH, the precipitate bears feature of chlorine-containing hydrous zirconia [20] rather than of an amorphous precursor of *t*-ZrO_2_. The latter phase appears as the only crystalline product after calcination at 500 °C. In Zr-1.5-800 the transformation of most of the tetragonal phase to *m*-ZrO_2_ is visible, although the intensity of the remaining 2Θ = 30.2° reflection of *t*-ZrO_2_ is stronger than in the Zr-1.0-800 counterpart. Its very weak trace persists even in Zr-1.5-1000, although monoclinic zirconia is the predominant crystalline phase found in this material.

**Figure 5:**
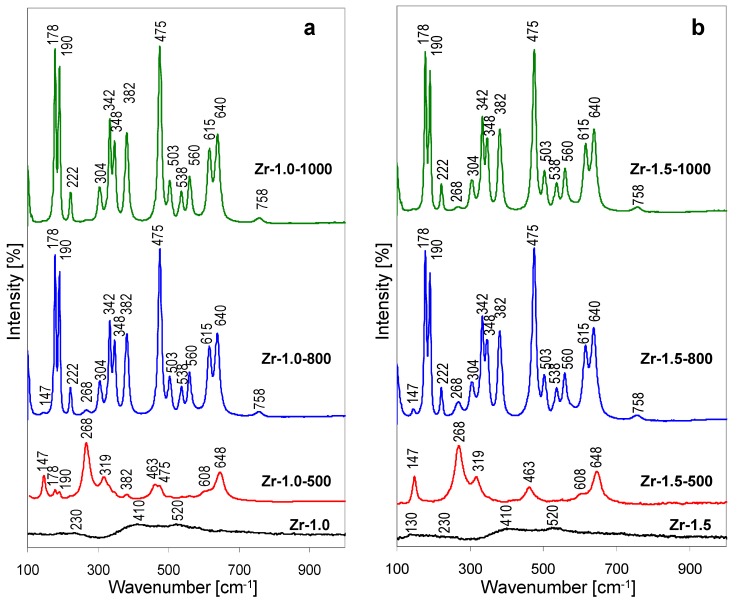
Raman spectra illustrating the effect of thermal treatment on hydrolysates obtained by lyophilization of Zr-pillaring solution: (**a**) As received at pH = 1.0, (**b**) after pH adjustment to 1.5.

The Raman spectra of the thermal evolution of Zr-1.0 sample are shown in Figure 5a, those recorded for the Zr-1.5 series, in Figure 5b. The spectra of Zr-1.0 and Zr-1.5 are quite similar and display several broad bands, centered around 130, 230, 410 and 520 cm^−1^. Similarly, as in the case of Ti-1.2, no bands attributable to NH_4_Cl impurity are visible. The observed features are similar to those reported previously for non-crystalline solids obtained from freshly hydrolyzed ZrOCl_2_ [22,23]. The intermediate character of Zr-1.0 and Zr-1.5 precipitates, referred to as chlorine-containing hydrous zirconia, is evidenced by the fact that the observed broad bands are different from those characteristics of ZrOCl_2_ hydrate precursor (460 and 590 cm^−1^) [21], but do not yet display features typical of zirconia polymorphs (see thermal evolution of Raman spectra). In contrast, the spectra of thermally treated Zr-1.0-500 and Zr-1.5-500 show well defined bands characteristic of tetragonal zirconia (147, 268, 319, 463, 608, 648 cm^−1^) [21,24]. In accordance with the XRD findings, the spectrum of Zr-1.0-500 shows some low intensity features characteristic of the most intense bands of *m*-ZrO_2_ (178, 190, 382, 475 cm^−1^). The Raman spectra recorded for samples treated at 800 and 1000 °C are similar, and reflect the transformation of most, or all of the calcined material to monoclinic ZrO_2_ polymorph. In agreement with XRD analysis, small amounts of *t*-ZrO_2_ are still visible in both samples calcined at 800 °C (low intensity bands at 147 and 268 cm^−1^), albeit with higher intensity in the Zr-1.5-800 sample. A trace of tetragonal zirconia can be still detected in the spectrum of Zr-1.5-1000 (weak band at 268 cm^−1^) bands, thus confirming that alkalization of Zr-pillaring solution yields hydrolysate in which tetragonal to monoclinic zirconia transformation is retarded. 

SEM images of samples Zr-0.1-1000 and Zr-1.2-1000, presented in Figure 3b,e, respectively, show that in both materials’ zirconia grains consist of variously shaped agglomerates. 

### 3.3. Hydrolysates Formed from Ti, Zr-Pillaring Solution

**Figure 6:**
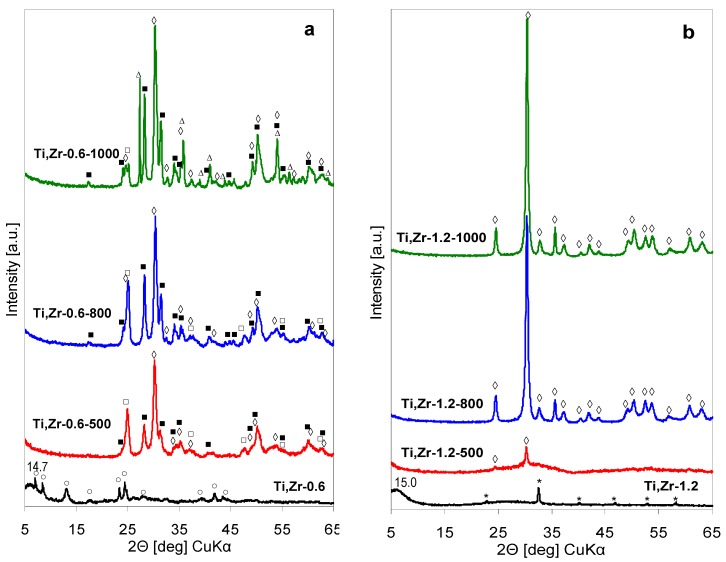
XRD patterns illustrating the effect of thermal treatment on hydrolysates obtained by lyophilization of Ti, Zr- pillaring solution: (**a**) As received at pH = 0.6, (**b**) after pH adjustment to 1.2 (○ ZrOCl_2_∙xH_2_O, ■ *m*-ZrO_2_, □ anatase, ◊ TiZrO_4_, ∆ rutile, * NH_4_Cl).

XRD patterns of precipitate recovered by lyophilization from the as received Ti, Zr-pillaring solution (pH = 0.6) and of the products of its thermal evolution are shown in Figure 6a. Those recorded for the materials obtained from the Ti, Zr-pillaring agent after adjustment of pH to 1.2, i.e., the value observed during Ti, Zr-PILC synthesis, are gathered in Figure 6b.

The XRD pattern of lyophilized Ti, Zr-0.6 precipitate shows a set of reflexes pointing to the presence of a certain amount of variously hydrated ZrOCl_2_. Formation of these phases shows that in the as received Ti, Zr-pillaring solution the tendency to form mixed species, if at all present, is limited. Moreover, the zirconyl chloride phases are better defined than in the case of Zr-1.0 precipitate obtained from the Zr-pillaring solution, which is attributed to the slower hydrolysis of the salt in the conditions of lower pH characteristic for mixed pillaring agent. The peaks of zirconyl chloride hydrates overlap with a component showing a broad maximum around 2Θ ≈ 6.0°, corresponding to an interplanar distance of ca. 14.7 Å, and an elevated background centered around 2Θ ≈ 28° (d ≈ 3.2 Å), indicating formation of a quasi-amorphous layered precursor of unknown composition. XRD analysis of sample calcined at 500 °C shows that the material is multiphase, with the evident presence of anatase form of titania, monoclinic polymorph of zirconia and mixed zirconium titanate (ZrTiO_4_). Moreover, in view of the strong overlap of XRD pattern of tetragonal zirconia with that of ZrTiO_4_, some contribution of the former cannot be completely excluded. The observed multiphase composition indicates that in the precipitate formed from the as received Ti, Zr-pillaring solution, there is a strong tendency to form separate Zr- and Ti-based phases.

**Figure 7:**
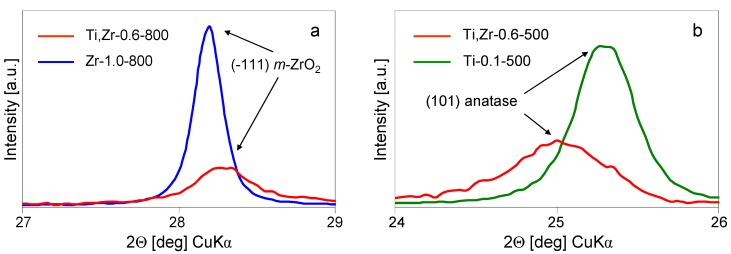
(**a**) Shift of (-111) XRD reflection of *m*-ZrO_2_ to higher 2Θ in multiphase Ti, Zr-0.6-800 with respect to its position in Zr-1.0-800, (**b**) shift of (101) XRD reflection of anatase to lower 2Θ in multiphase Ti, Zr-0.6-500 with respect to its position in Ti-0.1-500.

Calcination at 800 and 1000 °C temperatures confirms this observation, as separate phases appear to evolve independently (Figure 6a). Thus, the phase composition of the sample calcined at 800 °C is the same as after calcination at 500 °C, except for all phases showing better resolved reflections, consistent with their higher crystallinity. Noteworthy, in the case of material obtained from Zr-pillaring solution, the monoclinic form of ZrO_2_ is clearly visible only after calcination at ≥ 800 °C (see Section 3.2), while in the material obtained from Ti, Zr pillaring agent, *m*-ZrO_2_ appears already at 500 °C. Teng, et al. [25] reported that partial isomorphous substitution of Zr with Ti facilitates tetragonal to monoclinic polymorph transition. Indeed, in the present study a shift in the position of (-111) reflection of *m*-ZrO_2_ (indexed according to JCPDS 37-1484) to higher 2Θ values, with respect to monoclinic zirconia obtained from Zr-pillaring solution, is observed (Figure 7a). This points to a decrease of the corresponding interplanar distance and is consistent with a degree of substitution of large Zr^4+^ cations (ionic radius 0.72 Å) with smaller Ti^4+^ species (ionic radius 0.605 Å). After calcination at 1000 °C, the transformation of anatase to rutile is observed, albeit not complete, as some anatase can still be detected. This shows that phase transformation in titania obtained from mixed Ti, Zr-pillaring solution occurs at temperature ca. 200 °C higher than in the sample prepared from Ti-pillaring agent (see Section 3.1). Also, here the likely explanation is that in anatase formed from Ti, Zr-pillaring solution some Ti is substituted with Zr, the effect known to slow down the anatase to rutile transformation [25,26,27,28]. Indeed, the (101) reflection of anatase (indexed according to JCPDS 21-1272), observed in the sample obtained from mixed Ti, Zr-pillaring solution, is shifted to slightly lower 2Θ value (Figure 7b), which is consistent with the partial replacement of Ti^4+^ with larger Zr^4+^ ions.

XRD patterns of solids obtained from pillaring solution whose pH was adjusted to 1.2 with ammonia addition (Figure 6b), reveal a completely different nature of lyophilized Ti, Zr-1.2 precipitate and of products of its thermal treatment at 500, 800 and 1000 °C. Apart from the sharp reflections, due to formation of some NH_4_Cl, the Ti, Zr-1.2 precursor has a quasi-amorphous character. The broad peak at 2Θ ≈ 5.9° (d ≈ 15.0 Å) points to the nucleation of a layered type of structure, whose other feature is an elevated background around 2Θ ≈ 28° (d ≈ 3.2 Å). In Ti, Zr-1.2-500 the low 2Θ reflection disappears, but the sample is still strongly amorphous, except for two peaks emerging from the broadly elevated background, assignable to the most intense reflections of ZrTiO_4_. Full set of reflections characteristic of zirconium titanate becomes visible after calcination at 800 and 1000 °C. Thus, in contrast to the multiphase nature of solids evolving from Ti, Zr-0.6 sample, ZrTiO_4_ is the only phase crystallizing from the quasi-amorphous Ti, Zr-1.2 precipitate. 

**Figure 8:**
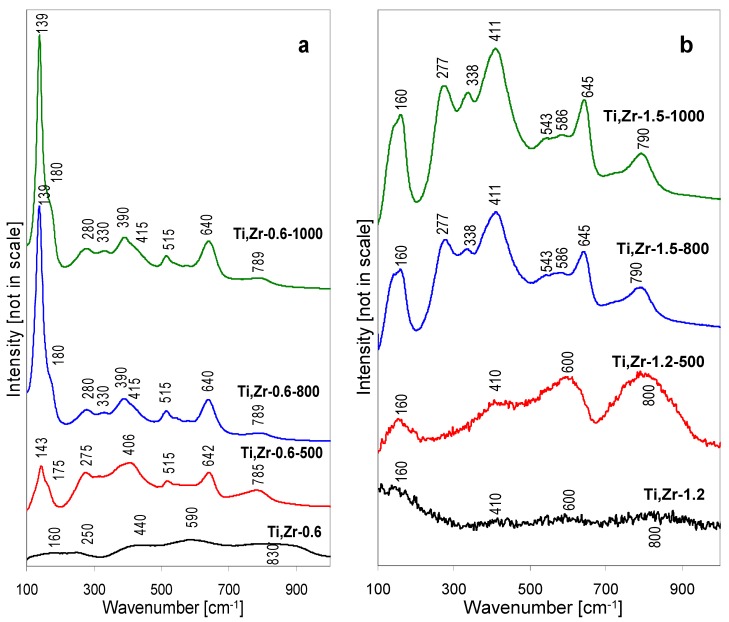
Raman spectra illustrating the effect of thermal treatment on hydrolysates obtained by lyophilization of Ti, Zr-pillaring solution: (**a**) As received at pH = 0.6, (**b**) after pH adjustment to 1.2.

The Raman spectra of Ti, Zr-0.6 and Ti, Zr-1.2 series of samples are gathered in Figure 8a,b, respectively. The spectrum of lyophilized Ti, Zr-0.6 precipitate shows only very weak, broad bands. The features around 440 and 590 cm^−1^ may be attributed to zirconyl chloride hydrates, whose presence has been identified by XRD, the other, around 160, 250, and 830 cm^−1^, are assigned to the quasi-amorphous layered structure. It is likely that the amorphous phase contains both Ti and Zr, because the band at ca. 830 cm^−1^ has been previously attributed to the formation of Ti-O-Zr in amorphous Zr-rich oxide-like material [29]. The Raman spectra of Ti, Zr-0.6 sample calcined at 500, 800 and 1000 °C confirm the XRD findings as to the multiphase nature of these solids (Figure 8a).

Thus, after thermal treatment at 500 °C, the Ti, Zr-0.6-500 sample shows several bands, of which the ones at 143, 406, 515 and 642 may be attributed to anatase. Formation of zirconium titanate component is also documented by Raman spectrum. According to literature, the most intense Raman bands of this phase are found at ~160, ~275, ~340, ~410, ~640, and ~790 cm^−1^, weaker ones at ~540, 590 cm^−1^ [12,29,30,31,32,33]. The bands tend to be quite broad, which has been attributed to the random distribution of Zr and Ti atoms at equivalent structural positions, combined with anionic defects and/or minor variation of stoichiometry in polycrystalline materials [29,34]. The bands around 275 and 785 cm^−1^ may be taken as diagnostic for this compound, as they do not overlap with vibrations stemming from other phases. The ZrTiO_4_ modes at 410 and 640 cm^−1^ contribute to the intensity of 406 and 642 cm^−1^ maximum. The shoulder at 170 cm^−1^ may also be attributed to ZrTiO_4_. However, no Raman bands of monoclinic ZrO_2_, whose presence is documented by XRD, can be detected, even in the spectral ranges where no overlap is expected (e.g., lack of feature around 475 cm^−1^, very strong in *m*-ZrO_2_). As demonstrated by Livraghi, et al. [35], the scattering properties of ZrO_2_ are much poorer than those of anatase, hence its bands are easily obscured in the systems containing both components. After calcination at 800 and 1000 °C the overall character of the Raman spectra is preserved, except that the main features become more pronounced, due to the better crystalline order of the sample components. Noteworthy, the ~140 cm^−1^ band of anatase is also the strongest on the spectrogram of a sample calcined at 1000 °C, where, as evidenced by X-ray data, most of the anatase has been transformed into rutile, which confirms the exceptionally strong scattering properties of this modification of titania. For the same reason no rutile bands can be clearly distinguished in the spectrum of Ti, Zr-0.6-1000, even the least overlapping mode around 450 cm^−1^.

Raman spectra of samples obtained from titanium-zirconium pillared solutions with pH raised to 1.2, i.e., the value observed during pillaring of montmorillonite, are different (Figure 8b). The spectrum of the lyophilized Ti, Zr-1.2 precipitate is poorly resolved, showing, similarly to other thermally untreated materials, only very broad bands, barely elevated from the background, at ca. 160, 410, 600 and 800 cm^−1^. As in other materials whose pH has been adjusted with ammonia addition, the amount of NH_4_Cl formed in the precipitate is not sufficient to mark its presence in the Raman spectrum. Changes observed upon thermal treatment show a close relation between the ensuing spectra, as the bands do not disappear but gradually become better resolved to yield the spectrum characteristic of ZrTiO_4_. Thus, in the Ti, Zr-1.2-500 sample the faint features visible in the spectrum of Ti, Zr-1.2 become more intense, in accordance with the XRD data which show that in this material the crystallization of ZrTiO_4_ from amorphous phase has just begun. The Raman spectra of Ti, Zr-1.2-800 and Ti, Zr-1.2-1000 show the full set of bands expected for ZrTiO_4_ (160, 277, 338, 411, 543, 586, 645 and 790 cm^−1^). No bands that might indicate the presence of other oxide components are present, which confirms the single-phase nature of the material produced from Ti, Zr-1.2 precipitate. Moreover, the obvious kinship between the spectra is an indication that already in the thermally untreated quasi-amorphous precursor the short order interatomic bonding is closely related to that developed in the zirconium titanate. 

**Figure 9:**
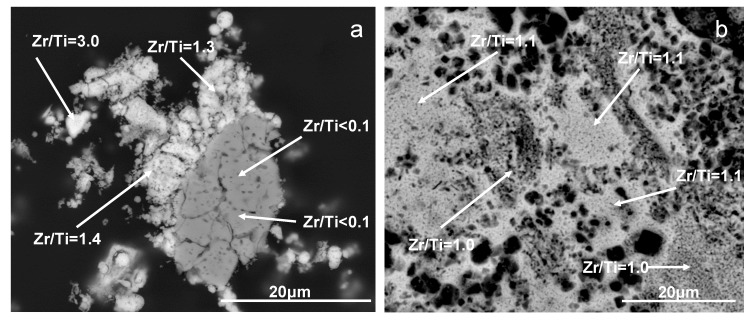
Scanning electron microscope (SEM)/Backscattered electrons (BSE) images of (**a**) Ti, Zr-0.6-500 and (**b**) Ti, Zr-1.2-500 samples. The Zr/Ti values found with EDS analysis for different areas of the samples are marked. Polished specimens of materials embedded in epoxy resin have been used for analysis.

SEM analysis of Ti, Zr-0.6-1000 and Ti, Zr-1.2-1000 samples reveals significant differences in morphology of both materials (Figure 3c,f, respectively). Thus, Ti, Zr-0.6-1000 is composed of grains with various shapes, ranging from small rounded particles to larger rectangular blocks. The inhomogeneous composition of Ti, Zr-0.6 series has been independently confirmed by the results of scanning electron microscope/energy dispersive spectrometer (SEM/EDS) analysis. Backscattered electrons (BSE) imaging, which strongly depends on the atomic number of the scattering elements, has been employed for analysis of the samples. BSE images are particularly suitable for detecting areas of different composition because the sample components containing heavier elements appear brighter. An example of the BSE image of Ti, Zr-0.6-1000 is shown in Figure 9a. The polycrystalline material is clearly composed of areas of different contrast, indicating differences in chemical composition of various parts of the specimen. The EDS analysis confirms that brighter regions are zirconium-rich, while darker contain mainly titanium. On the other hand, EDS analysis of Ti, Zr-1.2-1000 sample shows that the material is essentially homogeneous as far as the chemical composition is concerned, and no meaningful fluctuations around the expected Ti/Zr ratio equal 1 are observed (Figure 9b). The results support the conclusion drawn on the basis of the XRD and Raman data, that the mixed oxide material obtained from Ti, Zr-1.2 precursor is structurally and compositionally uniform. 

Comparison of the data obtained for materials synthesized from mixed Ti, Zr-0.6 and Ti, Zr-1.2 precursors points to the critical influence of the pH of the solutions on the nature of mixed oxide materials derived from the lyophilized precipitates upon thermal treatment. For the former series an inhomogeneous polycrystalline material is produced, with the tendency to form, next to zirconium titanate, separate Ti- and Zr-based phases. On the contrary, in the solids prepared from Ti, Zr-1.2, the ZrTiO_4_ phase, accommodating both elements, is formed exclusively. Since the pH value of 1.2 has been selected to imitate the acidity generated during preparation of Ti, Zr-pillared montmorillonite, the results of the current experiments back the conclusion presented in our earlier work [2] that pillars possess a TiZrO_4_-like nature rather than constitute a mixture of titania and zirconia props.

## 4. Conclusions

Results of the present study show that even a relatively moderate alteration of pH in Ti-, Zr-, or Ti, Zr-precursor solutions, such as that occurring during clay pillaring, brings about a substantial change in the outcome of hydrolytic transformations, manifested by different phase and/or chemical composition of hydrolysates recovered from these solutions. In particular, alkalization facilitates transformation of anatase into rutile in materials obtained from Ti-pillaring solution, but delays conversion of tetragonal to monoclinic zirconia in samples derived from Zr-pillaring agent. The most striking effect is observed for the mixed Ti, Zr-solution, where the increase of pH is the necessary condition for the formation of a single-phase material with mixed Ti-O-Zr network, rather than an inhomogeneous multiphase precipitate with the tendency to form separate Ti- and Zr-based phases obtained from the more acidic precursor. This finding indirectly supports the model of mixed titanium-zirconium props in pillared montmorillonites, and is an important indication for the design of Ti-Zr mixed oxide materials dedicated for other applications.
